# PReS-FINAL-2063: Proposed criteria for activity, damage and impact of juvenile idiopathic arthritis associated uveitis: consensus effort from the multinational interdisciplinary working group for uveitis in childhood (MIWGUC)

**DOI:** 10.1186/1546-0096-11-S2-P75

**Published:** 2013-12-05

**Authors:** I Foeldvari, A Heiligenhaus, J Anton, R Ramanan, C Edelsten, T Saurenmann, B Bodaghi, K Kotaniemi, S Nielsen, E Rabinovich, Multinational Interdisciplinary Working Group for Uveitis in Childhood (MIWGUC)

**Affiliations:** 1 Hamburger Zentrum für Kinder-und Jugendrheumatologie, Hamburg, Germany; 2 Ophthalmology, Müster, Germany; 3 Pediatric Rheumatology, Barcelona, Spain; 4 Pediatric Rheumatology, Bristol, UK; 5 Great Ormond Street Hospital, London, UK; 6 PediatricRheumatology, Zürich, Switzerland; 7 Ophthalmology, Paris, France; 8 Ophthalmologist, Helsinki, Finland; 9 Pediatric Rheumatology, Copenhagen, Denmark; 10 Pediatric Rheumatology, Durham, USA

## Introduction

Uveitis is the most common extraartricular manifesation of juvenile idiopathic arthritis (JIA) with an.incidence of approx. 10 - 15 per 100,000 children. Uveitis, a potentially blinding disorder, is present in approx 10-18% of JIA patients, with a wide spectrum of presentations. Despite advances in treatment of childhood arthritis there are no definitions for assessment of activity, damage and impact of JIA associated uveitis.

## Objectives

The purpose of our efforts was to develop and gain consensus on such definitions as activity and damage.

## Methods

At the second consensus meeting of the MIWGUC group, using a Delphi method we developed definitions for activity, damage and impact of disease based on already published outcome measures [1].

## Results

The following items were included and listed in Table 1. The items were derived from the previously proposed and already published list of outcome measures[1]. Following items were selected to present activity: anterior chamber cells, flare, vusual acuity, syncechiae,macular edema ocular hyptony, ocular hypertension, CHAQ, number of medication and local eyedrops. Folowing items were selected to present damaga:flare, visual acuity, synechiae,cataract, macular edema, ocular hypotony and hypertony, glaucoma, disc edema, band keratopahty, epiretinal membranes and overall uveiits disability VAS.

## Conclusion

We will validate these proposed definitions propsectively in a JIA associated uveitis cohort. Based on the results, we will weight these measures to develop an overall scoring system.

## Disclosure of interest

None declared.
